# Supplementary material for: Use of admission serum neutrophil gelatinase-associated lipocalin (NGAL) concentrations as a marker of sepsis and outcome in neonatal foals
Source: PLoS One. 2023 May 18;18(5):e0285819. doi: 10.1371/journal.pone.0285819 (PMC10194986; doi:10.1371/journal.pone.0285819)
Supplement: Attachment — Submitted filename: Response to reviewers.docx [file pone.0285819.s002.docx]

Dear Reviewers,

We kindly thank you for your comments for the manuscript intitled “***Use of serum neutrophil gelatinase-associated lipocalin (NGAL) concentrations as a marker of sepsis and outcome in hospitalized neonatal foals on admission*** ”. All comments will be addressed in the below rebuttal letter.

Kind regards,

Malene Laurberg

as representative for all co-authors of the manuscript

Journal Requirements:

When submitting your revision, we need you to address these additional requirements.

1. Please ensure that your manuscript meets PLOS ONE's style requirements, including those for file naming. The PLOS ONE style templates can be found at
   [https://journals.plos.org/plosone/s/file?id=wjVg/PLOSOne_formatting_sample_main_body.pdf](https://protect-au.mimecast.com/s/6hH9CBNqjlCV6L35rhjeVX5?domain=journals.plos.org) and 
   [https://journals.plos.org/plosone/s/file?id=ba62/PLOSOne_formatting_sample_title_authors_affiliations.pdf](https://protect-au.mimecast.com/s/XXIACD1vlpTBv8QlzSl2xOC?domain=journals.plos.org)

Authors reply: Please find the adapted parts in the track changes.

2. Please note that PLOS ONE uses a single-blind peer review procedure. We would therefore be grateful if you could include in the information that has been redacted (line 75) for peer review in the manuscript.

Authors reply: Please find the redacted information now available in the manuscript (line 77).

3. In your Methods section, please provide additional details regarding participant consent from the owners of the animals. In the ethics statement in the Methods and online submission information, please ensure that you have specified (1) whether consent was informed and (2) what type you obtained (for instance, written or verbal). If the need for consent was waived by the ethics committee, please include this information.

Authors reply: The need for consent was waived by the ethics committee. See lines 80-81 in the manuscript.

4. Please include your full ethics statement in the ‘Methods’ section of your manuscript file. In your statement, please include the full name of the IRB or ethics committee who approved or waived your study, as well as whether or not you obtained informed written or verbal consent. If consent was waived for your study, please include this information in your statement as well.

Authors reply: Consent was waived by the ethics committee. Please see the added ethics subsection in track change (lines 80-81).

5. We note that you have referenced (ie. Bewick et al. [5]) which has currently not yet been accepted for publication. Please remove this from your References and amend this to state in the body of your manuscript: (ie “Bewick et al. [Unpublished]”) as detailed online in our guide for authors
   [http://journals.plos.org/plosone/s/submission-guidelines#loc-reference-style](https://protect-au.mimecast.com/s/O-DXCE8wmrtW9ZK52HQjOQW?domain=journals.plos.org)

Authors reply: The mentioned article is not found in our reference list.

6. Please include captions for your Supporting Information files at the end of your manuscript, and update any in-text citations to match accordingly. Please see our Supporting Information guidelines for more information: [http://journals.plos.org/plosone/s/supporting-information](https://protect-au.mimecast.com/s/b-2NCGv0oyCJYzo5Rt1Ox42?domain=journals.plos.org).

Authors reply: There is no ssupporting information for the tables or figures present in the manuscript.

[Note: HTML markup is below. Please do not edit.]

Reviewers' comments:

Reviewer's Responses to Questions

**Comments to the Author**

1. Is the manuscript technically sound, and do the data support the conclusions?

The manuscript must describe a technically sound piece of scientific research with data that supports the conclusions. Experiments must have been conducted rigorously, with appropriate controls, replication, and sample sizes. The conclusions must be drawn appropriately based on the data presented.

Reviewer #1: Partly

Reviewer #2: Yes

2. Has the statistical analysis been performed appropriately and rigorously?

Reviewer #1: No

Reviewer #2: No

3. Have the authors made all data underlying the findings in their manuscript fully available?

The [PLOS Data policy](https://protect-au.mimecast.com/s/x-zRCJyBrGf8rjPEvFykgGw?domain=plosone.org) requires authors to make all data underlying the findings described in their manuscript fully available without restriction, with rare exception (please refer to the Data Availability Statement in the manuscript PDF file). The data should be provided as part of the manuscript or its supporting information, or deposited to a public repository. For example, in addition to summary statistics, the data points behind means, medians and variance measures should be available. If there are restrictions on publicly sharing data—e.g. participant privacy or use of data from a third party—those must be specified.

Reviewer #1: Yes

Reviewer #2: Yes

4. Is the manuscript presented in an intelligible fashion and written in standard English?

PLOS ONE does not copyedit accepted manuscripts, so the language in submitted articles must be clear, correct, and unambiguous. Any typographical or grammatical errors should be corrected at revision, so please note any specific errors here.

Reviewer #1: Yes

Reviewer #2: Yes

5. Review Comments to the Author

Please use the space provided to explain your answers to the questions above. You may also include additional comments for the author, including concerns about dual publication, research ethics, or publication ethics. (Please upload your review as an attachment if it exceeds 20,000 characters)

Reviewer #1: The sample size of the study is rather small. Report confidence intervals for sensitivity and specificity and discuss power in discussion.

Authors reply: The small sample size has been added to the discussion on study limitations (Line 347-349). Confidence intervals for the specificity and sensitivity of NGAL are added.

It seems based on the background information on other available methods, there isn’t much of a benefit to using this new biomarker? How do sensitivity and specificity compare? Discuss in methods why this method is worth pursuing further.

Authors reply: there are clear indications that NGAL also functions as an inflammatory marker, and as such we thought it interesting to test it as marker for the main inflammatory condition in foals: sepsis. Other available methods to diagnose sepsis have major limitations, and low diagnostic capabilities. Therefore, identifying new biomarkers that could be of use to confirm sepsis is clinically relevant. We believe this is clearly explained in the introduction, and we feel it is incorrect to provide further explanations in the methods section, as the methods section is supposed to explain what we do, not why we do it. Given the high specificities of NGAL for predicting sepsis and non-survival (90.0 % and 95.2 % respectively), NGAL could be used to rule out the presence of sepsis and the risk of non-survival.

It is true that the low sensitivity for NGAL to predict the presence of sepsis and the risk of non-survival (52.4 % and 39,3 % respectively) does pose a limitation. The diagnostic accuracy could potentially be increased by combining NGAL with other biomarkers or by doing repeated measurements.

Objectives in the introduction don’t quite match the title. The title suggests you are predicting both sepsis and outcome.

Authors reply: Title has been slightly altered to better fit the objectives of the study. **“Use of serum neutrophil gelatinase-associated lipocalin (NGAL) concentrations as a marker of sepsis and outcome in hospitalized neonatal foals on admission”**.

What was the range and median of days from admission to discharge/death? And clarify there was no follow-up after discharge? (from my understanding)

Authors reply: Unfortunately, we did not collect data on the length of hospitalization for this study. No follow-up post discharge was available, and this is noted in the methods.

Summarize the amount of missing data for the variables in the sepsis score calculation.

Authors reply: There was an acceptable level of missing data for the sepsis score: an average of 9.9 out of the 14 parameters were obtained. 62.6 % of foals had available data for ≥ 10 sepsis score parameters. This has been added to the text under results.

There were few foals with missing data for the survival score: an average of 5.9 out of 6 parameters were obtained. 98.9 % of foals had available data for ≥5 survival score parameters. This has been added to the text under results.

L55: what data exactly?

Authors reply: We are not sure what the reviewer means by this. Line 55 is a part of the introduction and does not refer to any data.

It states that statistical analyses were not conducted due to small sample size for comparing sepsis severity, but I recommend merging some of the smaller groups for purpose of comparison. For example, you could group severe sepsis with septic shock.

Authors reply: Subgroups severe sepsis and septic shock have now been grouped for the purpose of statistical comparison.

Add ROC curves so ranges of sensitivity and specificity can be seen.

Authors reply: ROC curves have been added. Please see new figures 3 and 6. Adaptions have been made with track changes to the text.

L303-304: But age was not addressed in this study even though it was collected? I understand the low sample size, but reporting descriptive statistics stratified by the foal characteristics you collected would be a good baseline for future work to be based on.
Since NGAL has not been previously reported on in foals, it would be interesting to summarize NGAL values by the other data collected. If there are correlations with other biomarkers and if NGAL is different based on other characteristics (including age).

Authors reply: No apparent difference in NGAL values are present over different ages (trend observation). This has been added to the manuscript. We fully understand that the reviewers are interested in a comparison between NGAL and creatinine as NGAL has been found a biomarker for AKI. This however has not been the aim of this study, but we are currently working on a second paper that assesses the use of NGAL as renal marker in the same population of foals, and here it will be compared to creatinine. This is noted in the manuscript.

The 3rd figure is missing a label for x axis.

Authors reply: This has been adapted. Please see new fig 4.

Reviewer #2: General comments: This is a well-written manuscript describing differences in serum neutrophil gelatinase-associated lipocalin (NGAL) concentrations between septic and non-septic foals, as well as between surviving and non-surviving foals. As the authors acknowledge, availability of a serum biomarker that could predict both sepsis and survival would be useful to equine clinicians. Unfortunately, although NGAL concentrations were specific, they had relatively low sensitivity for either sepsis or survival. Consequently, it remains unclear whether NGAL will become a practical biomarker, as compared to currently used clinical and laboratory parameters used to support sepsis and to predict outcome. The authors acknowledge this problem in their discussion of limitations of the manuscript as they state that: “In future studies, diagnostic accuracy may be improved by combining NGAL with other markers or as an integral part of the sepsis score”. This statement also points out one of this reviewer’s main concerns with this manuscript: the authors have collected clinical and laboratory data from this cohort of foals, yet they have not made any attempt to compare NGAL to other clinical or laboratory data. The manuscript could be much stronger if this type of comparison would be made though multivariable linear regression analysis of the data collected; specifically, well-documented risk factors for sepsis (failure of passive transfer) or simple laboratory parameters that would support sepsis (leucocyte count, glucose concentration, others). As an example, the authors are referred to: Giguère S, Weber EJ, Sanchez LC. Factors associated with outcome and gradual improvement in survival over time in 1065 equine neonates admitted to an intensive care unit. Equine Vet J. 2017 Jan;49(1):45-50 – at the least this manuscript should be referenced in this manuscript.

Authors reply: We have chosen not to compare NGAL directly to the collected clinical and laboratory data as this data is already used as a part of the case definition for sepsis and survival scoring as explained in the materials and methods section of the manuscript. We do believe that the selected method is appropriate for the objectives stated in this manuscript. We are currently working on a second manuscript that evaluates NGALs ability to predict AKI in the same population of foals.

Next, it would be useful to have more information about the non-surviving foals: 1) how many died/were euthanized; 2) if euthanized – due to generalized sepsis (non-responsive flat foal) or localized sepsis (e.g., musculoskeletal system); 3) were any euthanized for congenital anomalies (would not be suspected to have increased NGAL); and 4) age at admission of survivors vs non-survivors (a previously documented risk factor). Further, if a foal was euthanized for localized musculoskeletal sepsis at 2-3 weeks of age but was otherwise healthy, NGAL concentrations might be expected to be lower than in 1-3-day-old flat foal with generalized sepsis.

Authors reply: Of the 91 foals, 25 were non-survivors and of them 23 were euthanised and 2 died naturally. Due to euthanasia caused by financial restraint 3 foals were excluded from this part of the study. For non-survivors cause of death was known in 40% of cases and ranged from generalised sepsis to severe septic shock. In 60% of non-survivors cause of death could not be fully established from clinical records, but no evidence exists that the cause was a congenital malformation This has been added to the test under the results section.

Another major limitation of this manuscript is the inability to discriminate between septic and non-septic foals as the largest group (n=62) was the uncertain sepsis status group. This problem could be considered a “fatal flaw’” of the study.

Authors reply: This has been addressed in the limitations section of our discussion. See lines 344-349. We believe that one of the reasons for the high number of foals in the uncertain sepsis status group is due to our very strict inclusion criteria in the study. These inclusion criteria are believed to be even stricter that most studies using sepsis as a case definition. It also shows the inherent difficulties of diagnosing sepsis.

Finally, since NGAL is also considered a biomarker of acute kidney injury (AKI), it would be of interest to compare NGAL concentrations to creatinine concentrations in these groups of foals.

Authors reply: We are currently working on a second paper where NGAL is assessed as a biomarker for AKI. This second paper will amongst others compare NGAL concentrations to creatinine. However, we have added creatinine values for the groups of this study and compared them over the groups.

Although I will leave the decision on acceptability of the manuscript as written to the Editor, I think this manuscript would be considerably strengthened if these points of concern would be addressed. As written, this manuscript would not provide much impetus for a commercial agency to attempt to develop a foal-side NGAL ELISA test.

Authors reply: We fully agree with the reviewers opinion that NGAL has not shown to be a strong marker for sepsis or outcome. Further research is needed to assess diagnostic capabilities of NGAL. However, we do believe that even negative findings are important to help guide further research.

There are also a few minor edits that should be addressed that are detailed below under specific comments should the authors pursue a further revision.

Specific comments:

Introduction
Line 70 – delete “the” before diagnosis
Authors reply: Please see track change to line 72 in the new manuscript.

Materials and Methods
Lines 96-98 – What is “heterologous determination”? Was NGAL measured in duplicate/triplicate? Was there any internal quality control samples for NGAL to determine variance in the clinical use of the assay?

Authors reply: All samples were run in duplicates. To the authors knowledge no quality control samples were added to establish variance. Inter- and intra-assay variability have previously been established to range between 8.12 % - 28.39 % and 5.35 % - 12.9 % respectively. See reference 8.

Results
Table 1. It would be of interest to include (n=#) in Table 1 for the initial 4 lines on how sepsis was documented – realizing that some foals would have more than one criterion to support sepsis; could then possibly eliminate lines 171-172

Authors reply: These numbers are all found in table 2.

Line 177 – suggest changing “seemed” to “trended”
Authors reply: Has been adapted. Please see track change to line 196.

Discussion
Line 226 – suggest changing “have never previously been” to “not been”

Authors reply: Has been adapted. Please se track change to line 272.

Lines 243-245 – again, the authors could examine the potential relation between AKI and sepsis in this group of foals

Authors reply: this was added to the manuscript. There was no statistical difference in creatinine values between septic and non-septic foals or between sepsis severity subgroups. Likewise there was no statistical difference in creatinine values between survivors and non-survivors.

Line 247 – it would be useful to define the nature of the “insult” as the proceedings cited [8] may not be widely available to treaders of this manuscript

Authors reply: Has been defined in the new manuscript. Please see track change to lines 294-295.

Lines 249-251 – the authors are stating the obvious – again raising the question why a multivariable approach was not pursued in this study

Authors reply: It was decided to use the sepsis scoring system to strengthen our sepsis and case definition. By using the sepsis scoring system directly as a part of our selection criteria we have included a partial comparison to multiple other diagnostic variables such as neutrophil count, fibrinogen, glucose concentration, arterial oxygen saturation and the presence of metabolic acidosis. However further comparisons to these variables are not believed to be correct, as the same variable should not be used in the case definition and in a comparison at the same time.

Line 257 – consider changing “capacity” to “accuracy”

Authors reply: This has been adapted. Please see track change to line 305.

Line 260 – delete “significantly” – unnecessary

Authors reply: Has been adapted. See line 307.

Line 268 – change “can” to “could”

Authors reply: This has been adapted. Please see track change to line 316.

Lines 275-276 – “differed between survivors and non-survivors and were inversely associated
with a survival score (lower serum NGAL concentrations with higher likelihood of survival” – this statement suggests a correlation analysis was performed – but there is no description of such an analysis in the Methods

Authors reply: No correlation was done. It is based on trend observation. This has now been added to the text. Please see track change to line 325.

References
Double check to confirm accuracy and consistency of presenting the references

Figures – should include some notation (asterisks or other marker) to indicate significant differences

6. PLOS authors have the option to publish the peer review history of their article ([what does this mean?](https://protect-au.mimecast.com/s/HHDqCK1DvKTqER05NH2YaDn?domain=journals.plos.org)). If published, this will include your full peer review and any attached files.


If you choose “no”, your identity will remain anonymous but your review may still be made public.


**Do you want your identity to be public for this peer review?** For information about this choice, including consent withdrawal, please see our [Privacy Policy](https://protect-au.mimecast.com/s/YLPaCL7EwMfPjz5nrf5-h6b?domain=plos.org).

Reviewer #1: No

Reviewer #2: **Yes:** Harold C. Schott II
