# Supplementary material for: Use of admission serum neutrophil gelatinase-associated lipocalin (NGAL) concentrations as a marker of sepsis and outcome in neonatal foals
Source: PLoS One. 2023 May 18;18(5):e0285819. doi: 10.1371/journal.pone.0285819 (PMC10194986; doi:10.1371/journal.pone.0285819)
Supplement: Attachment — Submitted filename: Response to reviewers.docx [file pone.0285819.s003.docx]

Dear Reviewer,

We thank you for your comments for the revised manuscript intitled “**Use of admission serum neutrophil gelatinase-associated lipocalin (NGAL) concentrations as a marker of sepsis and outcome in neonatal foals**”. All comments will be addressed in the below rebuttal letter.

Kind regards,

Malene Laurberg

as representative for all co-authors of the manuscript.

Reviewers' comments:

Reviewer's Responses to Questions

**Comments to the Author**

1. If the authors have adequately addressed your comments raised in a previous round of review and you feel that this manuscript is now acceptable for publication, you may indicate that here to bypass the “Comments to the Author” section, enter your conflict of interest statement in the “Confidential to Editor” section, and submit your "Accept" recommendation.

Reviewer #2: (No Response)

2. Is the manuscript technically sound, and do the data support the conclusions?

The manuscript must describe a technically sound piece of scientific research with data that supports the conclusions. Experiments must have been conducted rigorously, with appropriate controls, replication, and sample sizes. The conclusions must be drawn appropriately based on the data presented.

Reviewer #2: Yes

3. Has the statistical analysis been performed appropriately and rigorously?

Reviewer #2: No

4. Have the authors made all data underlying the findings in their manuscript fully available?

The [PLOS Data policy](https://protect-au.mimecast.com/s/Sz3LCmO5glu5YBoAMS9WHR5?domain=plosone.org) requires authors to make all data underlying the findings described in their manuscript fully available without restriction, with rare exception (please refer to the Data Availability Statement in the manuscript PDF file). The data should be provided as part of the manuscript or its supporting information, or deposited to a public repository. For example, in addition to summary statistics, the data points behind means, medians and variance measures should be available. If there are restrictions on publicly sharing data—e.g. participant privacy or use of data from a third party—those must be specified.

Reviewer #2: Yes

5. Is the manuscript presented in an intelligible fashion and written in standard English?

PLOS ONE does not copyedit accepted manuscripts, so the language in submitted articles must be clear, correct, and unambiguous. Any typographical or grammatical errors should be corrected at revision, so please note any specific errors here.

Reviewer #2: Yes

6. Review Comments to the Author

Please use the space provided to explain your answers to the questions above. You may also include additional comments for the author, including concerns about dual publication, research ethics, or publication ethics. (Please upload your review as an attachment if it exceeds 20,000 characters)

Reviewer #2: General comments: This is a well-written revised manuscript describing differences in serum neutrophil gelatinase-associated lipocalin (NGAL) concentrations between septic and non-septic foals, as well as between surviving and non-surviving foals. As the authors acknowledge, availability of a serum biomarker that could predict both sepsis and survival would be useful to equine clinicians. Unfortunately, although NGAL concentrations were specific, they had relatively low sensitivity for either sepsis or survival. Consequently, it remains unclear whether NGAL will become a practical biomarker, as compared to currently used clinical and laboratory parameters such as glucose and IgG concentrations to support sepsis and to predict outcome.

In their revised manuscript, the authors have addressed some of the concerns raised in my initial review of this manuscript. Importantly, they have added information about serum creatinine concentrations. Unfortunately, they failed to perform a more robust analysis of the data collected as suggested in my initial review. Rather, they refer to data being presented in another manuscript in preparation. Again, this is disappointing and makes the usefulness of this report questionable. At the least, it would have been useful to include information about current “biomarkers” of sepsis such as serum glucose and IgG concentrations on admission. If NGAL concentration performed no better than these already accepted biomarkers of sepsis and survival, there would seem to be little incentive to further investigate NGAL (not just because we can measure it).

**Authors reply:**

Correlations between NGAL and currently used biomarkers to predict sepsis such as WBC, IgG, glucose and SAA values were done through linear correlation and a Spearman non-parametric tests. No correlation between NGAL and WBC was found (p = 0.64 and p = 0.52 respectively). No correlation between NGAL and IgG was found (p = 0.18). No correlation between NGAL and glucose values was found (p = 0.16 and p = 0.47 respectively). A significant linear relation between NGAL and SAA was found (p = <0.001). As we do not believe that these comparisons of NGAL to WBC, glucose and IgG levels are helpful for the objectives of our study and because these parameters were used as a part of the case selection (for sepsis and survival) we have opted not to include these results in the final manuscript. However, we wanted to show you that we have done these analyses per your request.

In our opinion SAA would fit our objectives and SAA was not used as part of the case selection criteria. Therefore we have opted to include the comparison between NGAL and SAA in the manuscript. Thank you for this helpful suggestion. The correlation between NGAL and SAA was also seen in other studies that our research group has done on NGAL (published as a conference presentation) and support NGALs use as a marker of inflammation. This data along with the SAA concentrations for the different groups of foals can be found in Table 2. We believe that with this addition, we have provided a strong scientific comparison of NGAL between sepsis categories, especially considering the fact that we have made a big effort to categorize patients as correct as possible in the sepsis categories by the use of our robust case definition. The manuscripts’ objective is not to show that NGAL is a better biomarker than established ones; its objective is to see if there is a difference between sepsis categories to get an idea if there is potential for NGAL as marker for sepsis. Even if it would not show any differences, it would still be scientifically sound and worth of being communicated to the equine community, as it is important to share negative results to understand the biomarker. Now that it is established that there are differences between sepsis categories, a follow up study can compare different sepsis diagnostic tools, without including these tools in the case definition. Furthermore, whether a new biomarker will be practically / clinically useful or not, is never decided in only one single study; it depends on a thorough validation process including different steps of validation, and will need to be examined by multiple studies, and by multiple centers and on different case populations.

Next, the validity of NGAL measurements warrants a bit more discussion in methods and discussion. In the authors validation study, linearity was only assessed by dilutional parallelism to concentrations up to 600 µg/L (reference 8, Figure 1) and it appears there were only 2 samples with values of 2000-3000 µg/L (reference 8, Figure 3). However, in the current manuscript, NGAL values approaching 5000 µg/L are presented in the Figures – how do we know if these values are accurate? It would be useful for the authors to present a “range” for accurate assay performance in the Methods section of this manuscript as well as mentioning potential assay limitations in the Discussion. This point is important as values at the extreme ends of the data set can have a substantial influence on correlations and development of ROCs.

**Authors reply:**

Thank you for raising this point. This has now been addressed in the methods section and in the discussion.

Further, the authors suggest that one reason sensitivity might have been low could have been that admission NGAL concentrations had not reached maximal values in earlier stages of sepsis. However, the same limitation could also used for diagnostic accuracy. If foals had a low NGAL at admission, that subsequently increased as sepsis progressed, the “low” value at admission would suggest a high likelihood of survival that would have changed as sepsis progressed. This limitation should also be acknowledged in the Discussion.

**Authors reply:**

We do agree that biomarker kinetics of NGAL could affect the diagnostic and prognostic ability equally. As the reviewer states if NGAL potentially has not reached maximal values upon admission this could affect the ability to diagnose sepsis or predict survival. This could be ameliorated by using serial measurements within the first 24-48 hours of hospitalization. Most foals in this study had not had repeat bloodwork done during hospitalization and sadly therefore we were unable to test this theory. We have added a note in the discussion.

Once again, the authors state in their conclusion: “In future studies, diagnostic accuracy may be improved by combining NGAL with other markers or as an integral part of the sepsis score, or through use of repeated measurements.” It is a shame that they did not make the effort to perform these analyses in this manuscript since all the data was available to them.

**Authors reply:**

we have further clarified our decision on this topic in the discussion.

Consequently, the only novel aspect to this manuscript is that it is the first to report serum NGAL concentrations in foals. As such, this data is worthy of publication but this reviewer again remains disappointed it the lack of further analyses that could have been made with this data set

**Authors reply:**

We hope that with the data that was added to this revision, the reviewer will be more content.

There are also a number of minor edits that should be addressed that are detailed below under specific comments should the authors pursue a further revision.

Specific comments:

Title: suggest rewording to: Use of admission serum neutrophil gelatinase-associated lipocalin (NGAL) concentrations as a marker of sepsis and outcome in neonatal foals

**Authors reply:**

Please find this adapted in the new headline.

Abstract
Line 25 – delete “for”

**Authors reply:**

Please find this adapted in line 25.

Line 33 – suggest using another term for “normal” sepsis – a bit of an oxymoron as sepsis is certainly not normal

**Authors reply:**

Despite the fact that this is indeed a bit of an oxymoron, and we are certainly not debating that sepsis is not normal, we have not changed this. It is namely a specific sepsis subcategory. We need it to be clear to the reader that the subcategory “normal sepsis” is different to the main category “sepsis”.

Introduction
Line 48 - delete “most” before fatal

**Authors reply:**

Please find this adapted in line 50.

Line 53 – suggest replacing “results” with “evidence”

**Authors reply:**

Please find this adapted in line 55.

Lines 56-60 – combine sentences into a single paragraph

**Authors reply:**

Please find these sentences re-written into a single paragraph. Lines 58-61.

Line 62 – change “for use in horses” to “for equine serum”

**Authors reply:**

Please find this adapted in line 63.

Lines 68-69 - suggest changing “Knowledge of equine NGAL is sparse, and no information on NGAL in foals is currently available.“ to “Currently, no information about NGAL is available for foals.”

**Authors reply:**

Please find this adapted in lines 69-70.

Materials and Methods
Line 99 – can delete “standardly”

**Authors reply:**

Please find this adapted in line 100.

Line 123 – add “concentration” after glucose

**Authors reply:**

Please find this adapted in line 126.

Line 129 – change “(13). Including” to “(13), including” – otherwise sentence starting with “Including” is an incomplete sentence

**Authors reply:**

Please find this adapted in line 132.

Line 132 – add “to support a diagnosis of SIRS” to the end of the sentence

**Authors reply:**

Please find this adapted in line 135.

Line 135 – change “as explained” to “detailed”

**Authors reply:**

Please find this adapted in line 138.

Line 136 – again, change “normal” sepsis; also in Tables 1 and 2

**Authors reply:**

Please see our answer provided above.

Line 166 – “Spaerman” should be “Spearman”

**Authors reply:**

Please find this adapted in line 169.

Results
Line 196 – suggest changing “trended” to “tended”

**Authors reply:**

Please find this adapted in line 203.

Table 2. It would be preferable to list data as mean ± SD, followed by median (range)

**Authors reply:**

Please find this adapted in table 2.

Discussion
Line 292 – add a period after (19)

**Authors reply:**

Please find this adapted in line 306.

Line 293 – consider changing “capacity” to “accuracy”

**Authors reply:**

Please find this adapted in lines 306-307.

Line 296 – consider changing “sufficiently” to “maximally”

**Authors reply:**

Please find this adapted in line 310.

Line 348 – consider changing “selected” to “used” to limit repetition

**Authors reply:**

Please find this adapted in line 368.

References
Double check to confirm accuracy and consistency of presenting the references. Reference 3 has authors names in ALL CAPITAL LETTERS. Reference 25 has CAPITAL letters starting most words in the manuscript title

**Authors reply:**

Please find this adapted in the reference section.

Figures – should include some notation (asterisks or other marker) to indicate significant differences

**Authors reply:**

Please find this adapted in figures 1 and 4 including their legends.
