# Supplementary material for: Use of admission serum neutrophil gelatinase-associated lipocalin (NGAL) concentrations as a marker of sepsis and outcome in neonatal foals
Source: PLoS One. 2023 May 18;18(5):e0285819. doi: 10.1371/journal.pone.0285819 (PMC10194986; doi:10.1371/journal.pone.0285819)
Supplement: Attachment — Submitted filename: Response to reviewers.docx [file pone.0285819.s004.docx]

Dear Reviewer,

We thank you for your comments for the revised manuscript intitled “**Use of admission serum neutrophil gelatinase-associated lipocalin (NGAL) concentrations as a marker of sepsis and outcome in neonatal foals**”. All comments will be addressed in the below rebuttal letter.

Kind regards,

Malene Laurberg

as representative for all co-authors of the manuscript
Reviewers' comments:

Reviewer's Responses to Questions

**Comments to the Author**

1. If the authors have adequately addressed your comments raised in a previous round of review and you feel that this manuscript is now acceptable for publication, you may indicate that here to bypass the “Comments to the Author” section, enter your conflict of interest statement in the “Confidential to Editor” section, and submit your "Accept" recommendation.

Reviewer #2: (No Response)

2. Is the manuscript technically sound, and do the data support the conclusions?

The manuscript must describe a technically sound piece of scientific research with data that supports the conclusions. Experiments must have been conducted rigorously, with appropriate controls, replication, and sample sizes. The conclusions must be drawn appropriately based on the data presented.

Reviewer #2: Yes

3. Has the statistical analysis been performed appropriately and rigorously?

Reviewer #2: Yes

4. Have the authors made all data underlying the findings in their manuscript fully available?

The [PLOS Data policy](https://protect-au.mimecast.com/s/_MEiCjZ1N7iYnpXBWt7mcnt?domain=plosone.org) requires authors to make all data underlying the findings described in their manuscript fully available without restriction, with rare exception (please refer to the Data Availability Statement in the manuscript PDF file). The data should be provided as part of the manuscript or its supporting information, or deposited to a public repository. For example, in addition to summary statistics, the data points behind means, medians and variance measures should be available. If there are restrictions on publicly sharing data—e.g. participant privacy or use of data from a third party—those must be specified.

Reviewer #2: Yes

5. Is the manuscript presented in an intelligible fashion and written in standard English?

PLOS ONE does not copyedit accepted manuscripts, so the language in submitted articles must be clear, correct, and unambiguous. Any typographical or grammatical errors should be corrected at revision, so please note any specific errors here.

Reviewer #2: Yes

6. Review Comments to the Author

Please use the space provided to explain your answers to the questions above. You may also include additional comments for the author, including concerns about dual publication, research ethics, or publication ethics. (Please upload your review as an attachment if it exceeds 20,000 characters)

Reviewer #2: General comments: This well-written second revision addresses most of the concerns raised in my review of the first revision. However, they have not revised “normal sepsis” - an oxymoron. I suggest another categorization such as mild sepsis, although admittedly “mild” sepsis would also be challenging to define. I will let the Editor make a final decision on this concern.

Next, in response to my previous review, the authors did compare NGAL to other laboratory measures of inflammation – thanks for doing this. However, addition of SAA to the manuscript has raised another question. Specifically, was sensitivity and specificity of admission SAA concentration for sepsis, sepsis severity, and outcome better than NGAL. Since SAA can be measured stall-side, this would be an important comparison that could be included in this manuscript. Again, I do not want to further prolong review of this manuscript so I will defer to the Editor as to whether this additional analysis should be performed and included in a final revision of this manuscript.

**Authors reply:**

As we have previously stated we do agree that using the term “normal” sepsis is a bit of an oxymoron as sepsis certainly is not normal. But we also struggle to find a good replacement, and as we are all aware normal sepsis is certainly not always mild from a clinical point of view. We have however opted not to change this as it namely functions as a subcategory. To clarify this, we have added throughout the manuscript clearly whether we mean the subcategory normal sepsis or the main category sepsis throughout the manuscript so this should now be easily understandable to the reader. We will accept it if the editor wants us to change this.

The optimal cut-off value to predict sepsis using serum NGAL concentrations has been recalculated using only certain data, and thus excluding the cases with uncertain sepsis status as follows.

Using ROC curves and the calculation of the Youden indice it was possible to determine the optimal cut-off value to predict sepsis for both serum NGAL concentrations and serum SAA concentrations. For these calculations the group of foals with uncertain sepsis status were excluded as to only use certain data (healthy, sick non-septic or septic). This resulted in wide confidence intervals for both sensitivity and specificity because of low case numbers in each group.

For NGAL the optimal cut-off for predicting the presence of sepsis was 455 µg/L with a sensitivity of 71.4 % (95 % CI: 47.8 µg/L – 88.7 µg/L) and a specificity of 100 % (95 % CI: 68.7 µg/L – 100 µg/L). For SAA the optima cut-off for predicting the presence of sepsis was 62 mg/L with a sensitivity of 85.7 % (95 % CI: 63.7 mg/L – 97.0 mg/L) and a specificity of 87.5% (95 % CI: 47.4 mg/L – 99.7 mg/L).

Both serum biomarkers thus performed within the same ranges in this group of foals, however bigger studies with higher case numbers are needed to further compare the usefulness of NGAL and SAA to predict the presence of sepsis.

The reviewer has previously commented on the accuracy of NGAL measurements above the calibration curve (> 3000 µg/L). We agreed that samples above this range might be subject to inaccuracies, however after discussing this matter with the remainder of our research group we were made aware that this is not true. For samples with NGAL concentrations above the calibration curve further dilution was performed for them to fit within the calibration curve and reach a final concentration. To make the reader aware of this it has been added to the materials and methods section. Likewise, the section on inaccuracy has been removed from the discussion.

Specific comments:

Abstract
Line 33 – suggest using another term for “normal” sepsis – a bit of an oxymoron as sepsis is certainly not normal

**Authors reply:**

see our answer above.

Introduction
Line 63 – suggest changing “of” to “by” at end of line

**Authors reply:**

Please find this adapted at the end of line 62.

Materials and Methods
Lines 95-96 – need to add SAA here as well

**Authors reply:**

Please find this adapted at the end of line 94.

Line 132 – “Band” should be “band”

**Authors reply:**

Please find this adapted at the end of line 132.

Line 139 – again, change “normal” sepsis; also in Tables 1 and 2

**Authors reply:**

Please refer to previous answer on the same topic.

Discussion
Line 305 – add a period after (19)

**Authors reply:**

Please find this adapted in line 320.
